# Roles of Cartilage-Resident Stem/Progenitor Cells in Cartilage Physiology, Development, Repair and Osteoarthritis

**DOI:** 10.3390/cells11152305

**Published:** 2022-07-27

**Authors:** Wei Xu, Wei Wang, Da Liu, Dongfa Liao

**Affiliations:** 1 Trauma Centre, General Hospital of Western Theatre Command of PLA, Chengdu 610083, China; xuwei8716@163.com; 2 Department of Orthopaedics, General Hospital of Western Theatre Command of PLA, Chengdu 610083, China; wangweiheiyouying@163.com

**Keywords:** osteoarthritis, cartilage-resident stem/progenitor cells, superficial zone, proteoglycan 4, cartilage repair

## Abstract

Osteoarthritis (OA) is a degenerative disease that causes irreversible destruction of articular cartilage for which there is no effective treatment at present. Although articular cartilage lacks intrinsic reparative capacity, numerous studies have confirmed the existence of cartilage-resident stem/progenitor cells (CSPCs) in the superficial zone (SFZ) of articular cartilage. CSPCs are characterized by the expression of mesenchymal stromal cell (MSC)-related surface markers, multilineage differentiation ability, colony formation ability, and migration ability in response to injury. In contrast to MSCs and chondrocytes, CSPCs exhibit extensive proliferative and chondrogenic potential with no signs of hypertrophic differentiation, highlighting them as suitable cell sources for cartilage repair. In this review, we focus on the organizational distribution, markers, cytological features and roles of CSPCs in cartilage development, homeostasis and repair, and the application potential of CSPCs in cartilage repair and OA therapies.

## 1. Introduction

Articular cartilage covers the ends of long bone and is made up of hyaline cartilage. On the basis of histologic features, the adult articular cartilage consists of four zones: superficial, middle, deep, and calcified zones. The superficial zone (SFZ) is made up of small, flat cells expressing the lubricant lubricin (also known as proteoglycan 4 (PRG4)) to reduce joint motion friction. The middle zone consists of relatively small oval chondrocytes that are randomly oriented. The deep zone contains large round chondrocytes oriented in vertical columns perpendicular to the cartilage surface. Finally, beneath these top three zones lies the calcified zone, which consists of hypertrophic chondrocytes [1,2]. The structural and organizational features of cartilage cells along with collagen fibers are crucial for maintaining the function of articular cartilage [1].

As articular cartilage lacks nerve and blood vessel distribution, it has limited capacity for self-repair [3]. A local superficial cartilage injury is generally irreversible and gradually deteriorates to larger lesions over time, ultimately leading to osteoarthritis (OA). Both local cartilage injury and OA cause stiffness and disability of the joint, which seriously impair the patient quality of life [4,5]. Currently, there is no satisfactory therapeutic method for cartilage injury or OA, which imposes a serious burden on patients and society.

With intense advances in regenerative medicine and tissue engineering, cell-based approaches have been deployed over the last decades to facilitate cartilage repair [6]. The major challenges in cell-based cartilage repair include selection of the seeding cells, in vitro culture expansion, and chondrogenic differentiation of seeding cells [7,8]. Adult chondrocytes are prime candidates for cartilage regeneration, as chondrocytes are a resident cell type in cartilage [9]. As the number of chondrocytes is limited due to their low cell density in cartilage (1–10%), they must undergo in vitro culture expansion to obtain sufficient numbers before application [1]. However, culture-expanded chondrocytes are prone to dedifferentiating and losing their chondrogenic phenotype to form cartilage [10,11]. Alternatively, mesenchymal stromal cells (MSCs), including bone marrow MSCs, synovial MSCs, adipose tissue MSCs and so on, are considered preferable cell sources for cartilage repair in clinical studies because they are easy to isolate and possess multipotent differentiation capacity and a high proliferative capacity [3,12,13,14,15,16,17,18]. Despite their great potential to generate cartilaginous tissue, some studies have shown that MSCs tend to undergo terminal differentiation into hypertrophic chondrocytes and are subsequently replaced by osseous tissue [19,20]. These drawbacks could discourage further application of chondrocytes and MSCs for cell-based cartilage repair.

A distinct population of endogenous stem/progenitor cells that exists in the SFZ of articular cartilage, named cartilage-resident stem/progenitor cells (CSPCs), was isolated and identified in recent decades [21,22,23]. The critical in vitro characteristics of CSPCs include the colony-forming ability, multilineage differentiation potential (including chondrocytes, osteoblasts and adipocytes), and the expression of several MSC surface markers [24,25,26]. It has been reported that the closer the stem cells are to the damaged cartilage tissue, the more effective the cartilage repair is [27]. Contrary to MSCs, CSPCs do not acquire a hypertrophic phenotype in vitro during chondrogenic differentiation [28,29]. Therefore, CSPCs present in the SFZ of articular cartilage are a more ideal cell source for tissue engineering and cartilage defect repair than other cell types. In-depth and comprehensive studies on the function of CSPCs in articular cartilage development, adult homeostasis, and cartilage repair are vital for the development of novel therapeutic strategies for cartilage injury and OA.

## 2. Distribution of CSPCs

**Figure 1:**
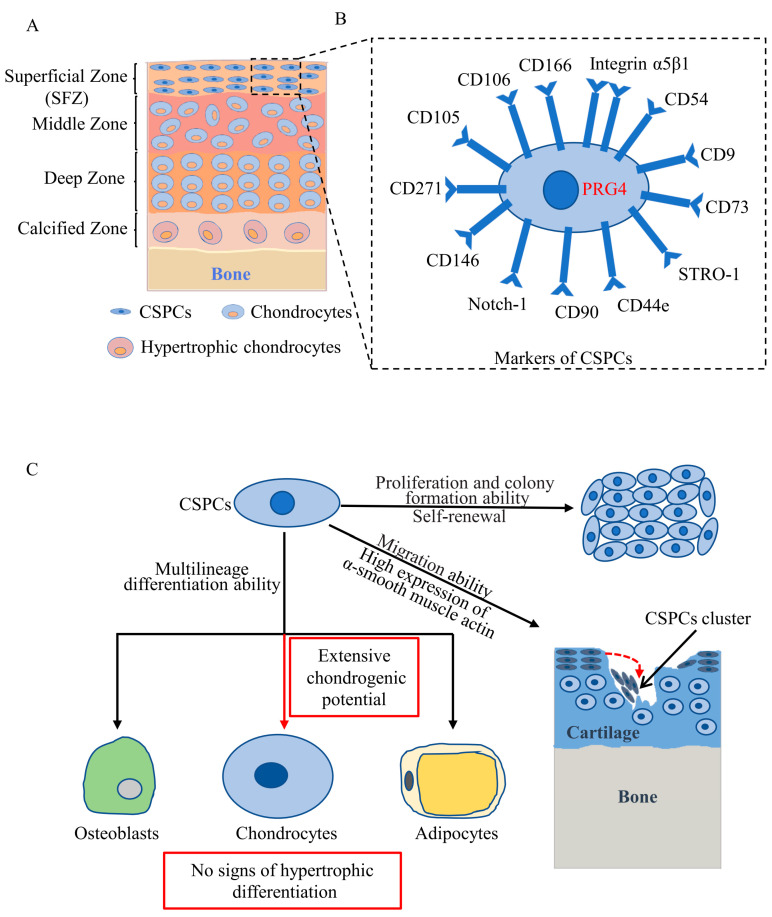
Zonal architecture of articular cartilage, including the distribution, markers and cytological features of CSPCs. (**A**) Normal adult cartilage consists of superficial, middle, deep, and calcified zones, and CSPCs reside in the superficial zone. (**B**) CSPCs express various MSC-related surface markers individually or in combination, and PRG4 is a specific marker of CSPCs in mice. (**C**) Properties of CSPCs: proliferative potential, colony-formation ability, multilineage differentiation ability and migration ability.

CSPCs have recently been discovered mostly in the SFZ of articular cartilage based on their features of expressing MSC surface markers, having multilineage differentiation ability, colony formation ability, cell quiescence, and migration ability in response to injury both in vitro and in vivo (Figure 1). Some studies have detected CSPCs in the SFZ of articular cartilage by exploring cell quiescence (very slow cycling) using bromodeoxyuridine or 5-ethynyl-2’-deoxyuridine pulse labelling, a defining property of stem cells. Initially, in 2001, Hayes et al. reported that articular SFZ cells had a longer cell cycle than the underlying chondrocytes and that articular cartilage growth is achieved by apposition from the SFZ [30]. Another independent study demonstrated that the SFZ of articular cartilage contains a slowly dividing stem cell (labelling retaining cells) population that replenishes the pool of cartilage cells through lateral and vertical expansion [31]. These studies laid the foundation for the presence of a population of stem/progenitor cells in the SFZ of articular cartilage that contributes to the appositional growth of the articular cartilage.

Taking advantage of the high expression of the fibronectin receptor (α5β1 integrin subunits), SFZ cells can be selectively isolated by differential adhesion to fibronectin. First, in 2004, Dowthwaite et al. [32] found that SFZ cells can form colonies in vitro and differentiate into cartilage, bone, tendon and muscle connective tissues in vivo and were therefore proposed to be CSPCs. Subsequently, numerous studies reported the expression of MSC-related markers and in vitro clonogenicity and multilineage differentiation of CSPCs in SFZ isolated by differential adhesion to fibronectin in mouse [33], rat [34,35,36], rabbit [37], bovine [38,39], equine [28], chicken [40], and human [41,42,43,44] articular cartilage. Alternatively, Grogan et al. [45] assessed the distribution of MSC markers neurogenic locus notch homologue protein 1 (Notch-1), STRO-1 and vascular cell adhesion protein 1 (VCAM-1, also known as cluster of differentiation 106 (CD106)), by immunohistochemistry and found that MSC markers were higher in the SFZ than in the deeper layer of articular cartilage, which indirectly confirmed the existence of CSPCs in the SFZ.

Elaborate cell-lineage tracing studies in mice that genetically trace the SFZ cell lineage in vivo provided powerful evidence for the existence of CSPCs in the SFZ. Three independent research teams revealed that SFZ cells expressing PRG4 are CSPCs, which contribute to the development of adult articular cartilage [21,22,23]. CSPCs in the SFZ of juvenile mice serve as progenitors for deeper-layer chondrocytes in adult mice [21,22,23].

Although most studies have demonstrated the existence of CSPCs in the SFZ, some studies have indicated that CSPCs may also reside in deeper layers of articular cartilage. Using fluorescence-activated cell sorting and clonogenicity screening, Yu et al. [46] isolated high-efficiency colony-forming CSPCs from both the SFZ and deeper layers of articular cartilage, and deeper layers of CSPCs possessed more chondrogenic and osteogenic capacity than SFZ CSPCs. Kozhemyakina et al. [21] found that some of the chondrocytes in the deeper layer of articular cartilage were PRG4 (a specific marker of CSPCs) positive in aged mice, which may be a sign that articular chondrocytes can dedifferentiate to CSPCs. Another study further supported the view that CSPCs can be isolated from mature human chondrocytes by adopting a low-density and low-glucose 2-dimensional culture system [47].

## 3. Markers of CSPCs

Currently, no specific stem cell surface markers that can be used to distinguish CSPCs from MSCs have been identified. CSPCs are generally isolated from tissue based on MSC-related surface markers, which can successfully distinguish them from chondrocytes. CSPCs are characterized by the traditional MSC markers individually or in combination, including CD105, CD73, CD54, CD106, CD166, CD9, CD90, CD146, CD44e, and CD271 (Figure 1B) [24,33,36,48,49,50,51,52,53,54,55,56]. Additionally, some studies have distinguished CSPCs in articular cartilage more specifically by using markers such as laminin, integrin-α5β1, Jagged, Notch-1, Notch-2, Delta, and STRO 1 [42,45,48,57,58,59]. However, none of the above studies identified a CSPC-specific marker to directly identify and distinguish them from MSCs and chondrocytes either in vitro or in vivo.

Employing elegant genetic tracing methods in mice, some studies have made breakthroughs in specifically identifying CSPCs in vivo. Initially, in 2015, Kozhemyakina et al. [21] knocked in a green fluorescent protein (GFP)-tamoxifen-inducible Cre recombinase (CreERT2)-expressing cassette into the endogenous *Prg4* gene in mice to visually trace the fate of SFZ cells (PRG4 is specifically expressed by SFZ cells and by cells lining the synovial membrane) in vivo. They demonstrated that PRG4+ cells presented in the SFZ of articular cartilage in young mice, which served as progenitors for cells both in the SFZ and deeper layer of the articular cartilage in aged mice and PRG4+ cells in the SFZ of young mice, can expand into deeper layers of the articular cartilage as the mice become older. Further study by Li et al. [23] showed that PRG4+ cells in the SFZ of articular cartilage are adult stem cells, which divide very slowly, are capable of self-renewal, express MSC surface markers and completely remodel adult articular cartilage. Decker et al. [22] further confirmed the above findings by employing two different transgenic mouse strains. In brief, these three studies confirmed that PRG4 is a specific marker of CSPCs that reside in the SFZ of articular cartilage in mice.

## 4. Cytological Features of CSPCs

The cytological features of CSPCs include extensive proliferative potential, intense chondrogenic potential with no signs of hypertrophic differentiation and migration in response to injury (Figure 1C) [59]. Numerous studies have demonstrated that CSPCs generally precede other cell types in proliferative ability in vitro [37,59,60]. The extensive in vitro expansion potential of CSPCs may overcome the disadvantage of limited cell sources of CSPCs. CSPCs have a proliferative potential for up to 60 population doublings when cultured in monolayers, and early-stage cultured cells have a colony-formation ability [38,39,41,61,62]. CSPCs were also reported to retain telomere length and telomerase activity after 20 population doublings, compared with chondrocytes [41]. Carluccio et al. [60] found that the division speeds of CSPCs were faster than those of chondrocytes. In contrast, Vinod et al. [44] reported that CSPC proliferation was slower than that of chondrocytes. Possible reasons for this inconsistency might be related to differences in isolation protocols and passage number of the cells between these studies.

The multilineage differentiation ability of CSPCs was characterized based on their ability to differentiate into chondrocytes, osteoblasts and adipocytes in vitro [28,29,34,37]. CSPCs isolated from osteoarthritic cartilage and subjected to colony forming assays were demonstrated to have chondrogenic, osteogenic, and adipogenic potential with variation in tri-lineage differentiation capacity between different clonal cell lines in vitro [62]. Furthermore, CSPCs maintained chondrogenesis potential after extensive expansion [41]. Tong et al. [34] found that the chondrogenesis potential of CSPCs was stronger than that of bone-marrow-derived MSCs. Li et al. [37] demonstrated that CSPCs have greater chondrogenic capacity than fat-pad-derived MSCs or chondrocytes cultured in alginate beads. Wang et al. [52] observed that CSPCs isolated from grade 3–4 OA cartilage exhibited increased osteo-adipogenic potential and decreased chondrogenic capacity, compared to CSPCs isolated from relatively normal cartilage. Su et al. [51] reported that CSPCs from late-stage OA cartilage displayed decreased adipogenesis and osteogenesis but increased chondrogenesis, compared to chondrocytes and adipose-derived stem cells. Furthermore, CSPCs did not express collagen type X after chondrogenic induction in vitro, contrary to MSCs [29].

Migratory ability facilitates CSPC migration to the cartilage defect site and participation in cartilage repair. SFZ cells express higher levels of α-smooth muscle actin than chondrocytes in deeper layers, which supports the idea that CSPCs have enhanced migratory ability [63]. Koelling et al. [64] showed that CSPCs derived from human articular cartilage are able to migrate >1 mm deep when cultured on the surface of later-stage OA cartilage. In addition, CSPCs can migrate to the site of injury after blunt impact or scratching in mature cattle osteochondral explants [65]. Platelet-derived growth factor BB (PDGF-BB) and insulin-like growth factor 1 (IGF-1) released from damaged cartilage facilitates CSPC migration, while interleukin 1 beta (IL-1β) and tumor necrosis factor alpha (TNF-α) decrease CSPC migration [66]. Recently, Wang et al. [67] reported that native resident CSPCs can migrate into a poly(lactic-co-glycolic) acid (PLGA) scaffold, and cultured human CSPCs seeded in PLGA scaffolds can migrate out from the scaffold in a rabbit model of osteochondral defects.

## 5. Roles of CSPCs in Articular Cartilage Development

**Figure 2:**
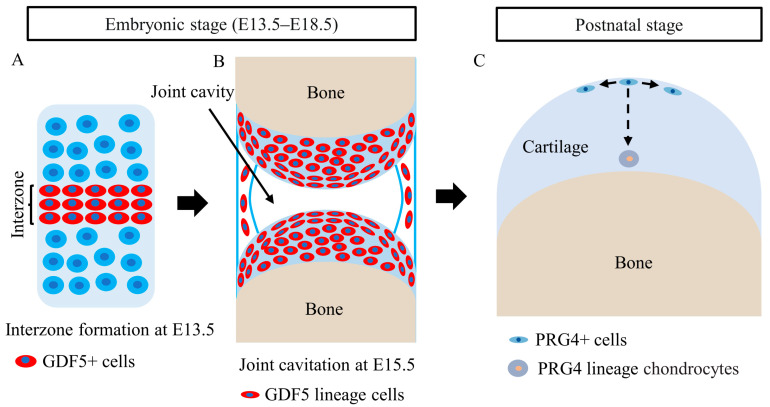
CSPCs in cartilage development. (**A**). GDF5+ cells constitute the interzone at the site of future joints. (**B**). During the embryonic stage, GDF5+ cells contribute to the formation of articular cartilage and other joint structures. (**C**). During the postnatal stage, PRG4+ cells in the SFZ are likely descendants of GDF5+ cells. PRG4+ cells in the SFZ are CSPCs that have self-renewing capacity to expand their population by symmetrical divisions, and PRG4+ cells in the SFZ give rise to the underlying layers of chondrocytes.

As the first indication of joint development, a condensed cell appearance at the site of future joints at E13.5 was defined as ‘‘interzone”. Subsequently, at E15.5, joint cavitation appears in the center of the interzone, and articular cartilage and synovial joints are gradually formed from interzone cells. During joint development, cells continuously efflux from the interzone to form articular cartilage, and the surrounding cells influx into the interzone to expand the interzone cell population [68]. Koyama et al. [69] reported that chondrocytes dedifferentiate to growth differentiation factor 5 (GDF5)+ cells and constitute the interzone at the site of future joints (Figure 2A). Numerous studies have demonstrated that GDF5+ cells contribute to the formation of articular cartilage and other joint structures (Figure 2B) [22,69,70]. Shwartz et al. proposed an influx model in which there is a continuous influx of mesenchymal cells from surrounding tissues into the interzone to expand GDF5+ cells and a simultaneous efflux of GDF5+ cells from the interzone to form articular cartilage [70]. Recently, Feng et al. [71] identified a subset of leucine-rich-repeat-containing G-protein-coupled receptor 5 (LGR5)+ cells in the GDF5+ cells of the interzone that contribute to the formation of articular cartilage and other joint structures. Moreover, they found that collagen XXII alpha 1 (COL22A1) was expressed by LGR5+ cells before the joint cavitation process, and the LGR5+/COL22A1+ cells were chondrocyte progenitors that contributed to forming the articular surface [71].

At the postnatal stage of articular cartilage development, CSPCs residing in the SFZ of articular cartilage contribute to all layers of articular cartilage (Figure 2C). By generating an inducible knock-in mouse model to lineage trace the descendants of PRG4+ cells in the SFZ, Kozhemyakina et al. [21] found that PRG4+ cells in the SFZ were CSPCs that gave rise to articular chondrocytes. When labeled at embryonic day 17.5 (E17.5), the daughter cells of PRG4+ cells extended as columnar clones from the SFZ into the deep layers beneath the tidemark in adulthood. The authors also proposed that articular cartilage was formed by the appositional growth, at which point CSPCs resident within the SFZ sequentially gave rise to the underlying layers of articular cartilage. Li et al. [23] further advanced this primary observation, showing that resident PRG4+ cells within the SFZ were CSPCs that divided slowly and gave rise to middle and deep zone chondrocytes. PRG4+ cells have a self-renewing ability and can expand their population by symmetrical divisions, with both progeny cells becoming CSPCs and remaining in the SFZ [23]. Furthermore, PRG4+ cells produced clonal clusters that facilitated both appositional and interstitial growth of articular cartilage in young mice and entirely reconstituted all layers of the adult articular cartilage [23]. Decker et al. [22] further confirmed these findings and showed that nondaughter cells stack with cell rearrangement contributing to articular cartilage growth and thickening, with limited contributions from cell proliferation but major contributions from zone-specific cell volume growth. Together, the above studies demonstrate that PRG4+ cells in the SFZ are CSPCs that contribute to articular cartilage development during postnatal life.

GDF5 marks the interzone domain during early embryonic development [70]. However, PRG4 marks the domain of CSPCs in the SFZ when joints were fully formed. Indeed, GDF5 expression first becomes restricted to the surface of articular cartilage at E14.5–E15.5 [70] and disappears from articular cartilage between E15.5 and postnatal day 0 (P0) [69,70,72]. Cells in the SFZ autonomously obtain PRG4 expression [69,72]. It is quite possible that these are the same cells that shut down GFD5 expression and turn on PRG4 expression [23]. In other words, PRG4+ cells in the SFZ are likely the descendants of GDF5+ cells [73].

## 6. Roles of CSPCs in Articular Cartilage Repair and OA

A large number of clues suggest that CSPCs may be involved in cartilage repair and OA. Seol et al. [65] observed that CSPCs migrated to the injury site 7–14 days after injury in a cattle explant trauma model. Multiple soluble factors released after cartilage injury regulate the migration of CSPCs, such as high mobility group protein B1 (HMGB1), IGF-1, PDGF and IL-1β [65,66,74,75,76]. Tong et al. [34] found that CSPCs were activated at the early stage of OA, but the number of activated CSPCs gradually decreased at the later stage of OA in a surgically induced OA model. Cell proliferation and MSC surface markers are increased in OA cartilage. Wang et al. [52] reported that more CD105+/CD271+ cells with higher expression of migratory ability-related molecules, such as LIM homeobox transcription factor 1-beta (LMX1B), SPARC-related modular calcium-binding protein 2 (SMOC2), and fibrillin-2 (FBN2), resided in OA cartilage than in relatively normal cartilage. In OA cartilage, the classical MSC surface markers CD105, VCAM-1, CD166, and CD146 were increased, individually or in combination [33,45,48,49,51,52,77]. During the early stage of OA, cell clusters were observed near the injury site of the cartilage, which expressed MSC surface markers, such as STRO-1, Notch-1 and VCAM-1 [45,78]. During the late stage of OA, Koelling et al. identified a progenitor cell population with stem cell characteristics that was positive for STRO-1 and CD29 in degenerated cartilage sites [64]. These findings suggest that CSPCs might be involved in OA pathogenesis and cartilage repair, but more elaborate research is needed to confirm this.

Currently, there is no satisfactory in vivo evidence that CSPCs play a role in cartilage repair or regeneration. PRG4+ cells simultaneously exist in the synovial membrane and in the SFZ of cartilage [21]. Decker et al. [22] demonstrated that the progeny of PRG4+ cells filled the injury site within 7 days after injury in an osteochondral defect model. Surprisingly, the progeny of PRG4+ cells in the synovium massively divide in response to injury, whereas neither PRG4+ cells nor their progeny in the SFZ near the injury site proliferate [22,73]. Similarly, Roelofs et al. [79] observed massive expansion of GDF5+ cells in the synovial membrane after acute cartilage injury but little proliferation of CSPCs in the SFZ near the injury site when genetically tracing GDF5+ cells in mice. These findings suggest that the progeny of PRG4+ cells in the synovium but not in the SFZ may contribute to cartilage repair or regeneration. In contrast, CSPCs in the SFZ were found to migrate to the site of injury in a cattle cartilage explant injury model [65]. We cannot rule out the possibility that CSPCs in the SFZ contribute to cartilage repair since the migration of CSPCs in the SFZ cannot be easily detected in genetically traced mice [73].

With the development of powerful genetic tools and the discovery of the CSPC-specific marker PRG4, direct evidence has been obtained for the role of CSPCs in OA pathogenesis. Loss-of-function mutation of *PRG4* in humans and *Prg4* knockout in mice result in early-onset arthropathy accompanied by a decreased number of CSPCs [80,81]. HMGB2 maintains the SFZ by supporting CSPC survival, while *Hmgb2* knockout mice exhibit enhanced cartilage degradation and develop OA [82]. Inducible β-catenin knockout in CSPCs results in enhanced OA development with impaired SFZ in adult mice, while β-catenin stabilization alleviates OA development [83]. Tan et al. observed accelerated senescence and decreased proliferation and differentiation of CSPCs accompanied by exacerbated OA development in CSPC-specific activin receptor-like kinase 5 [*Alk5*, also known as transforming growth factor beta receptor 1 (*Tgfβr1*)] knockout mice [84]. These findings suggest that CSPCs play significant roles in OA pathogenesis. In contrast, Zhang et al. [85] killed CSPCs through autonomous expression of diphtheria toxin in mice and demonstrated that CSPC death did not exacerbate OA development. Zhang’s findings seem to conflict with the opinion that CSPCs play significant roles in OA pathogenesis. Although in Zhang’s study most of the CSPCs were killed, there was evidence that surviving CSPCs could proliferate at a higher rate and partially restore their number [23,85]. Meanwhile, diphtheria induced CSPC ablation but did not physically injure the SFZ of articular cartilage, so this approach may not have been sufficient to activate a degradation process.

## 7. Signaling Pathways Regulating CSPCs during Articular Cartilage Development and OA

Numerous factors and signaling pathways have been found to play important roles in regulating CSPC homeostasis in the SFZ. PRG4 is produced by CSPCs and plays pivotal roles in the lubrication of joint surfaces and regulating the differentiation of CSPCs in the SFZ. Maenohara et al. [81] showed that the SFZ disappeared at eight weeks, accompanied by enhanced cartilage degeneration in *Prg4* knockout mice. *Prg4* knockout CSPCs displayed enhanced differentiation in vitro [81]. Mechanistically, PRG4 contributes to the homeostasis of articular cartilage by suppressing the differentiation of CSPCs, and the nuclear factor kappa-light-chain-enhancer of the activated B cells (NF-κB)-matrix metallopeptidase 9 (MMP9)-TGF-β pathway might be involved in this process [81]. Pin1 is a highly conserved peptidyl-prolyl isomerase involved in the regulation of stem cells. Zhang et al. [86] found that Pin1 is a critical regulator of CSPC senescence, as Pin1 protein levels are significantly reduced in senescent CSPCs and Pin1 knockdown by siRNA accelerated CSPC senescence.

Canonical Wnt/β-catenin signaling plays essential roles in the development and homeostasis of cartilage by regulating CSPCs in the SFZ. Wnt/β-catenin signaling has been found to be specifically activated in the SFZ of articular cartilage in adult mice [83]. Koyama et al. reported that conditional β-catenin knockout in collagen type II alpha 1 (COL2A1)- or GDF5-expressing cells resulted in SFZ defects [69]. Yasuhara et al. showed that Wnt/β-catenin signaling is essential for the proliferation and phenotypic expression of CSPCs in the SFZ, whereas β-catenin deficiency caused a loss of the CSPC population in the SFZ [87]. Inducible β-catenin knockout in CSPCs resulted in enhanced OA development with reduced expression of PRG4 and destruction of SFZ in adult mice, while β-catenin stabilization alleviated OA development with enhanced expression of the *Prg4* gene [83]. The activation of Wnt/β-catenin signaling with Wnt3a enhanced CSPC proliferation in vitro [87].

Epidermal growth factor receptor (EGFR) signaling also plays significant roles in the homeostasis and maintenance of CSPCs in the SFZ. EGFR signaling is dominantly activated in the SFZ of healthy articular cartilage of humans and mice, while it is remarkably inhibited in the SFZ of OA cartilage [88]. Cartilage-specific *Egfr* knockout impaired articular cartilage development and dramatically accelerated cartilage degeneration in a surgically induced OA mouse model [88]. EGFR signaling plays crucial roles in maintaining CSPC numbers in the SFZ and stimulates PRG4 secretion [88]. Cartilage-specific overexpression of EGFR in mice led to an expanded pool of CSPCs in the SFZ with enhanced proliferation ability and PRG4 production [89]. EGFR overactivation relieved cartilage damage and other signs of OA in a surgically induced OA model [89]. Taken together, these studies confirm EGFR signaling as a vital regulator of CSPCs in the SFZ during articular cartilage development and OA development.

In addition to these signaling pathways, TGF-β/ALK5 signaling has been identified as a regulator of CSPCs in the SFZ. Tan et al. [84] reported that conditional *Alk5* knockout in CSPCs resulted in accelerated OA development in spontaneous and surgically induced OA mouse models. *Alk5* knockout aggravated the progression of senescence and inhibited the proliferation and differentiation of CSPCs [84]. TGF-β/ALK5 signaling might upregulate PRG4 expression through the cyclic adenosine monophosphate (cAMP)-dependent protein kinase (PKA)-cAMP response element-binding protein (CREB) pathway [90].

Several transcription factors also play significant roles in the homeostasis and maintenance of CSPCs in the SFZ. HMGB2 is a transcriptional regulator that is specifically expressed in the SFZ of human articular cartilage, and its expression declines with age [82]. HMGB2 maintains CSPC survival, and HMGB deficiency leads to accelerated OA development [82]. Forkhead box O (FoxO) transcription factors are required for maintenance of stem cell populations. Cartilage-specific triple *FoxO* knockout led to early onset of OA in mice. The CSPC population and PRG4 expression were significantly decreased in triple *FoxO* knockout mice, while ectopic FoxO1 expression increased PRG4 production in vitro [91]. Zhang et al. [92] showed that CREB5 was a transcription factor that is uniquely expressed in CSPCs of SFZ and was essential for TGF-β and EGFR signaling to induce PRG4 expression. Yes-associated protein and transcriptional coactivator with PDZ-binding motif regulates PRG4 and tenascin C expression, acting as downstream effectors of cell division control protein 42 and actin polymerization in regulating the phenotype of CSPCs in the SFZ [93].

## 8. Application of CSPCs in Cartilage Regeneration and OA Therapies

CSPCs obtained from articular cartilage are an ideal cell source for cartilage regeneration, as these cells exhibit a high degree of chondrogenic capacity, extensive proliferative potential and apparent resistance to hypertrophy. CSPCs demonstrated higher chondrogenic potential than infrapatellar fat pad-derived stem cells and chondrocytes when cultured in alginate beads under intermittent hydrostatic pressure stimulation [37]. When cultured in 3D cell-laden hydrogel constructs, CSPCs generated more neocartilage than chondrocytes, and CSPCs expressed more collagen type II and PRG4 than MSCs [28]. CSPCs accumulated more cartilage-like tissue with good mechanical properties than perichondrium stem cells in a collagen scaffold system in vivo [94]. Hypoxic adipose-derived stem cell-derived extracellular vesicles promoted CSPCs to produce more cartilage matrix and proteoglycan in alginate hydrogel cultures [95]. Human CSPCs exhibit extensive chondrogenic potential, as evidenced by increased glycosaminoglycan production, upregulated cartilaginous marker gene expression and lack of hypertrophic marker expression when cultured in fibrin-polyurethane composite scaffolds with mechanical stimulation [96].

Several studies have applied CSPCs for in vivo cartilage regeneration. Williams et al. [41] demonstrated that CSPC-seeded scaffolds have an excellent ability to form cartilage-like repair tissue with good lateral integration with the near cartilage in a chondral defect model in goats. Mancini et al. [97] applied CSPCs and MSCs in a zonal composite osteochondral implant and revealed significant bone growth into the implant after six months in an equine model. Cui et al. [98] designed a novel injectable nanocomposite through integration of a chitosan-based hydrogel, CSPCs and mesoporous SiO2 nanoparticles loaded with anhydroicaritin. The injectable nanocomposites exhibited extensive CSPC proliferation and chondrogenic potential in vitro and an excellent ability to form cartilage-like repair tissue with good lateral integration with native cartilage in a cartilage defect model in rabbits [98]. Wang et al. [67] demonstrated that human-derived CSPCs seeded in PLGA scaffolds induce hyaline-like cartilage formation in a rabbit model of osteochondral defects. Finally, the clinical repair of large cartilage defects with CSPCs achieved satisfactory results. Jiang et al. [47] designed a pilot clinical study in 15 patients with large knee cartilage defects (6–13 cm^2^) and demonstrated that CSPCs have an excellent cartilage formation ability.

Some molecules that directly target CSPCs in the SFZ may be applied to OA therapy. As mentioned above, PRG4 is a specific marker of CSPCs in the SFZ, which also plays pivotal roles in lubrication of joint surfaces and regulating the differentiation of CSPCs [80]. The intra-articular injection of PRG4 derived from human synoviocytes increased PRG4 expression in the SFZ and alleviated cartilage degradation in a rat surgically induced OA model [99]. The small molecule 4-aminobiphenyl promotes cartilage regeneration by increasing the number of CD44+/CD105+ CSPCs and preventing matrix loss [100]. Intra-articular injections of 4-aminobiphenyl relieved OA progression in OA mice [100]. 

During the last decades, bioscaffolds seeding with cells have been largely applied to tissue regeneration [101]. Biotechnological chondroitin is a novel glycosaminoglycan which is eminently suitable for design of bioscaffolds [102]. Biotechnological chondroitin immensely increased human chondrocyte cell proliferation and was able to preserve human chondrocyte phenotype for a long time in vitro [103]. Biotechnological chondroitin was more effective in reducing the OA related inflammatory response and modulating the secretome profile compared to chondroitin sulfate [103,104]. Vassallo demonstrated that biotechnological chondroitin improved the mechanical properties of gelatin-methacryloyl based hydrogels and facilitated the differentiation of MSCs [102]. Taken together, these studies suggest that biotechnological chondroitin based bioscaffolds together with CSPCs may be of great value for application in cartilage regeneration.

## 9. Conclusions

Accumulating evidence suggests that CSPCs reside in the SFZ of articular cartilage, which is characterized by the expression of MSC surface markers, colony formation ability, multilineage differentiation ability, and migration ability in response to injury. In vivo cell lineage tracing studies have demonstrated that PRG4 is a specific CSPC marker in mice. PRG4+ cells present in the SFZ of articular cartilage contribute to articular cartilage development and homeostasis during postnatal life. CSPCs’ extensive proliferative and chondrogenic potential with no signs of hypertrophic differentiation highlight them as suitable sources for progenitor cell-based cartilage repair. However, CSPC numbers and chondrogenic potential progressively diminish with aging. Finding approaches for the prevention of CSPC senescence and the rejuvenation of senescent CSPCs could be of considerable value for the application of CSPCs in cartilage repair. In addition, investigating the role of CSPCs in OA pathogenesis and cartilage repair and finding biophysical or pharmaceutical factors to guide them to sites of injury are critical for designing intrinsic reparative therapies for OA.

## Data Availability

Not applicable.
